# Ensuring greenhouse gas reductions from electric vehicles compared to hybrid gasoline vehicles requires a cleaner U.S. electricity grid

**DOI:** 10.1038/s41598-024-51697-1

**Published:** 2024-01-18

**Authors:** Madalsa Singh, Tugce Yuksel, Jeremy J. Michalek, Inês M. L. Azevedo

**Affiliations:** 1 Department of Energy Science and Engineering, Stanford University, Stanford, USA; 2 Faculty of Engineering and Natural Sciences, Sabanci University, Istanbul, Turkey; 3 Smart Mobility and Logistics Lab, Sabanci University, Istanbul, Turkey; 4 Department of Mechanical Engineering and Department of Engineering and Public Policy, Carnegie Mellon University, Pittsburgh, USA; 5 Precourt Institute for Energy, Stanford University, Stanford, USA; 6 Woods Institute for the Environment, Stanford University, Stanford, USA; 7 Nova Business School, Carcavelos, Portugal

**Keywords:** Sustainability, Energy and society, Energy justice, Energy policy

## Abstract

Emissions from electric vehicles depend on when they are charged and which power plants meet the electricity demand. We introduce a new metric, the critical emissions factors (CEFs), as the emissions intensity of electricity that needs to be achieved when charging to ensure electric vehicles achieve lifecycle greenhouse gas emissions parity with some of the most efficient gasoline hybrid vehicles across the United States. We use a consequential framework, consider 2018 as our reference year, and account for the effects of temperature and drive cycle on vehicle efficiency to account for regional climate and use conditions. We find that the Nissan Leaf and Chevy Bolt battery electric vehicles reduce lifecycle emissions relative to Toyota Prius and Honda Accord gasoline hybrids in most of the United States. However, in rural counties of the Midwest and the South, power grid marginal emissions reductions of up to 208 gCO_2_/kWh are still needed for these electric vehicles to have lower lifecycle emissions than gasoline hybrids. Except for the Northeast and Florida, the longer-range Tesla Model S battery-electric luxury sedan has higher emissions than the hybrids across the U.S., and the emissions intensity of the grid would need to decrease by up to 342 gCO_2_/kWh in some locations for it to achieve carbon parity with hybrid gasoline vehicles. Finally, we conclude that coal retirements and stricter standards on fossil fuel generators are more effective in the medium term at reducing consequential electric vehicle emissions than expansion of renewable capacity.

## Introduction

The U.S. transportation sector in the United States accounts for 29% of the total greenhouse gas emissions (GHGs), with almost 60% of transport GHG emissions coming from light-duty vehicles^1^. A recent report by the National Academy of Sciences underscores the need to expand the adoption of electric vehicles. It highlights that electric vehicles (EVs) must become 50% of new vehicle sales by 2030 to achieve decarbonization goals^2^. Replacing gasoline vehicles with electric vehicles—which operate on electricity stored in rechargeable batteries—has enormous potential to reduce greenhouse gas emissions.

Life-cycle analysis (LCA) has been used extensively to compare the emissions implications of different vehicles. Usually, a life-cycle analysis (cradle-to-grave) includes vehicle and battery manufacturing emissions, vehicle use and associated fuel or carrier emissions, and disposal. LCA can be used in attributional or consequential frameworks^3,4^. The attributional approach focuses on the emissions and impacts associated with an activity based on modeler judgments of how to assign emissions to activities or products. For electricity-related emissions, for example, one would use the average power grid emissions per unit of energy calculated as the total emissions from electricity produced divided by the total electricity produced in a region. A consequential approach is used when focusing on outcomes that will *change* in response to a change in activity, such as adopting an EV instead of a gasoline vehicle or implementing an efficiency standard*.* For electricity, typically, marginal power grid emissions should be considered under this framework.

Existing LCA studies comparing EVs to other vehicles differ considerably regarding their scope of analysis and the inclusion (or omission) of some important factors. While all life-cycle stages are relevant, the use-phase emissions are most important, thus requiring a spatially explicit framework since EVs will be only as clean as the grid used to charge them. Studies have found that the emissions implications of EVs have substantial regional variation^3–17^ and depend on which vehicle types are being considered^5^. Other important factors influencing use-phase emissions include temperature, as that will effect vehicle energy consumption and HVAC^6–9^, the kind of road and driving^10–13^, and when/how the electric vehicles are charged^14–18^, as they may alter power plant dispatch, systems peak load, marginal generators, and associated emissions^15,19–28^. Tamayo et al.^29^ found that using average vs. marginal emission factors and the regional boundaries and methods used to estimate emission factors can substantially change electric vehicle emissions estimates. Yuksel et al.^14^ show that absolute and comparative life-cycle emissions are highly heterogeneous and depend on the choice of vehicles, ambient temperature, drive cycle, and charging profiles. Our work is closely related to Yuksel et al.^14^ and relies on the same life-cycle scope and incorporation of the same factors in our analysis.

Here, we develop a new metric, the Critical Emissions Factor (CEF), which we define as the carbon intensity of the regional grid that would be needed for the lifecycle emissions from an electric vehicle to be at parity with the emissions of some of the most efficient gasoline vehicles in the market. We do so while accounting for regional and operational aspects that lead to different emissions levels in different locations, such as climate (ambient temperature) and driving conditions. We also compare CEF to current grid emissions intensity to ascertain which regions have the highest GHG-reduction potential for the near-term widespread roll-out of electric vehicles.

## Methods and data

We estimate the lifecycle emissions for electrified vehicles and some of the most efficient gasoline hybrid vehicles in the United States. Our scope of analysis includes vehicle manufacturing (body of the vehicle and battery production for electric vehicles) and vehicle use. The use-phase emissions include emissions associated with the combustion of gasoline and emissions related to electricity production needed to run the vehicle. The use-phase emissions also include the upstream emissions from the production of the fuel or energy carrier (for both gasoline and electricity). Importantly, our assessment explicitly includes driving conditions, ambient air temperature, HVAC use, and make-model-specific energy consumption. We then estimate the electricity emissions intensity required for electrified vehicles to be at emissions parity with conventional vehicles.

We consider five representative vehicles for hybrid gasoline (HEV) and battery electric technologies (BEV) based on the vehicle class and availability of detailed laboratory test data assessing vehicle efficiency across multiple ambient temperatures and drive cycles. Two gasoline hybrids—Toyota Prius and Honda Accord Hybrid—were chosen to reflect the most efficient gasoline vehicles in the market, and three battery electric vehicles—Tesla Model S, Chevrolet Bolt, and Nissan Leaf—in decreasing battery capacity and, by extension, their range and weight (Table 1).

**Table 1:** Representative vehicles considered and their characteristics.

Vehicle model	Type, model year	Nameplate (usable) battery capacity	Weight (kg)
Tesla model S*	BEV, 2017	100 (95)	2230
Chevrolet bolt*	BEV, 2018	60 (57)	1616
Nissan leaf*	BEV, 2018	40 (36)	1570
Honda accord hybrid^+^	Gasoline HEV, 2015	–	1600
Toyota prius^+^	Gasoline HEV, 2010	–	1384

Data sources: *AAA; ^+^ANL.

**Figure 1:**
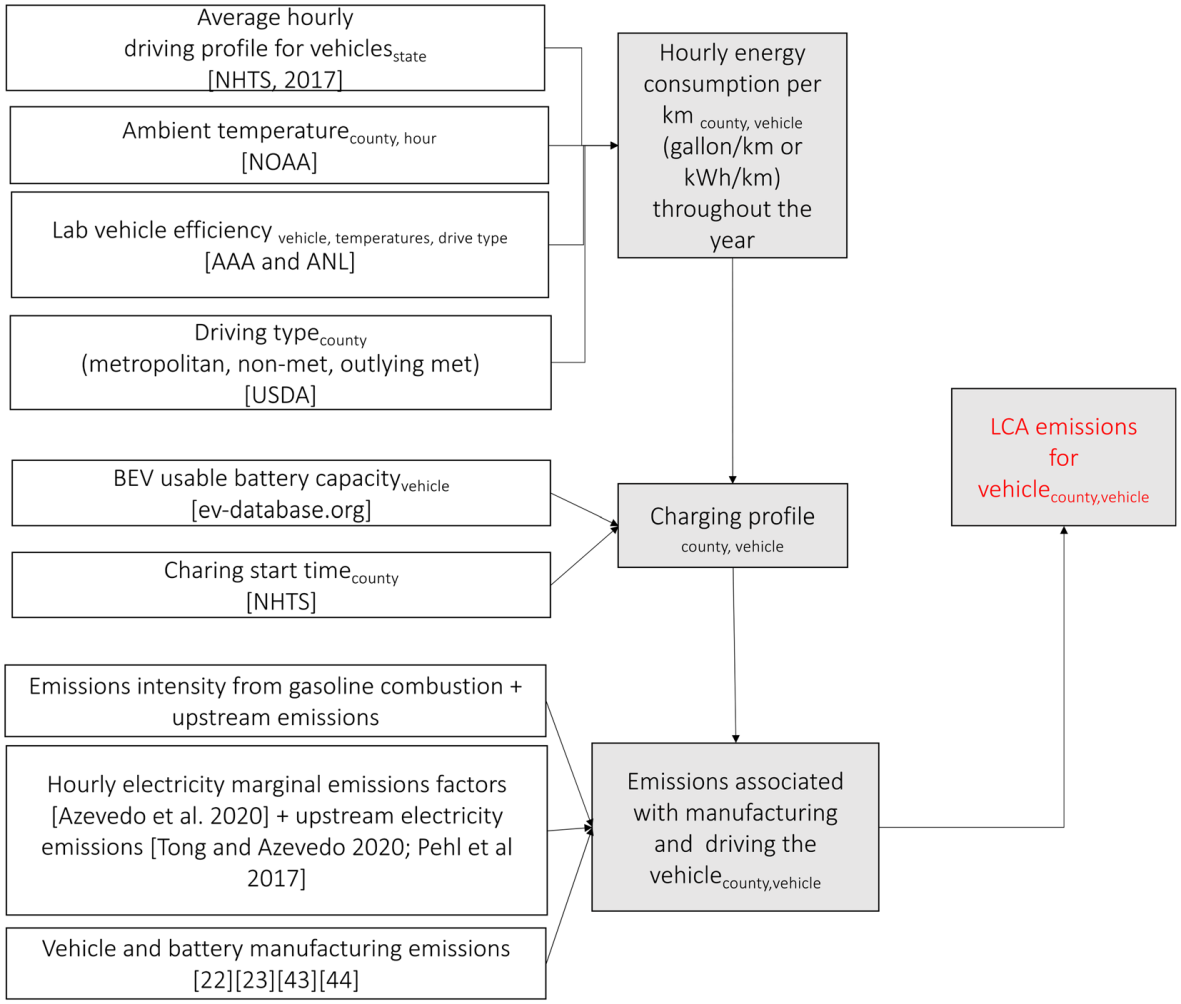
Modelling strategy and data sources.

Figure 1 describes our modeling strategy and data sources, which are as follows:

(i) *Driving and charging regimes* We use trip-level and vehicle-level information for 256 thousand vehicles from the 2017 National Household Transport Survey (NHTS) for private light-duty vehicles^30^. We incorporate weights for trips, households, and vehicles to reflect the geography where each household was initially sampled. Two inputs are derived from these data: First, we use trip-level data to build an average driving profile for each state, which shows *when* trips are taking place. This helps us incorporate those hours’ temperature effects in our energy consumption analysis. For instance, some states drive more in the morning and evening (California, Texas), while others show reasonably constant driving throughout the day (Montana, Maine). Second, we use vehicle and trip level data to build a driving matrix for each vehicle that includes the distance traveled by a vehicle in a day and the time of travel throughout the day. This matrix is used to identify a convenience-charging profile, which assumes that a vehicle will be charged after the last trip of the day. While NHTS 2017 has specific data for EVs, we use driving characteristics of all vehicles to derive charging profiles, as the sample for EVs is small and geographically concentrated, and we expect that mainstream driving patterns will be more relevant as electric vehicle adoption increases. Recent literature demonstrates that EVs are driven less than conventional vehicles in the U.S. Still, for this study, we assume similar driving distances and patterns for BEVs and HEVs^31^. A typical level-2 charger of 7 kW power and 85% charging efficiency is assumed.
(ii) *Temperature data* We use hourly on-ground monitoring station temperature measurements from NOAA’s Integrated Surface Database for 3000 stations in the contiguous United States for 2018^32^. Stations with more than 7000 data points (out of 8760 hourly values) were selected, and missing values were imputed using the value from the previous or next day for the same hour. This data allows us to incorporate temperature impacts in vehicle energy consumption.
(iii) *Laboratory data on vehicle energy consumption* We use the fuel economy of vehicles at three temperatures (− 6 °C, 25 °C, 35 °C) and for two standard EPA’s test-driving cycles (Urban Dynamo-meter Driving Schedule (UDDS), US06 (high acceleration), and Highway Fuel Economy Test (HWFET))^33^. During the tests at − 6 °C and 35 °C, the climate control is set to keep the cabin temperature at 25 °C through HVAC use. We use UDDS and HWFET tests to represent city and highway driving, respectively, and a linear combination (0.55 UDDS + 0.45 HWFET) represents the combined drive cycle. We use linear interpolation for other temperatures between − 6 and 35 °C. For temperatures lower and − 6 °C and 35 °C, we use the fuel economy for those temperatures. Dynamometer data for EVs comes from the AAA^34^, and data for gasoline vehicles (hybrid and conventional) is from Argonne National Lab^36^. Although data sources for EVs and HEVs differ, the same standardized certification drive cycles (as outlined by EPA) were used to reflect urban, highway, and combined driving.

Both testing agencies (ANL and AAA) conducted UDDS followed by HWFET as part of a test drive schedule at the same three temperatures to obtain vehicle fuel and energy consumption. Different instruments and machinery from the two testing agencies may introduce systemic differences between EVs and HEVs, but using standardized drive cycles will help reduce such confounders. Since the Toyota Prius and Honda Accord Hybrid data is derived from older vehicles, the fuel economy of older and 2018 models were compared from the Environmental Protection Agency’s (EPA) reported city, rural, and combined fuel economy values from fueleconomy.gov^37^. A 2018 Toyota Prius has marginally higher fuel economy values compared to a model from 2010 (city mpg: 54 (2018) vs. 51 (2010), rural mpg: 50 vs. 48, and combined mpg: 52 vs. 50), while a 2018 Honda Accord Hybrid has similar fuel economy compared to its 2015 model (city mpg: 48 (2018) vs. 49 (2015), rural mpg: 47 vs 47, and combined mpg: 48 vs 45). Wherever applicable, values for 2-cycle tests were proportionally increased by corresponding fuel economy ratios.

However, these tests are known to produce optimistic fuel consumption results relative to on-road driving, resulting in lower than actual emission estimates^35^. Thus, we have run sensitivity analyses using 5-cycle tests for HEVs, which include drive cycles to reflect aggressive driving (US06) and driving with extreme temperatures (SC03) along with urban and highway driving. For EVs and HEVs, we estimate and use the *derived* 5-cycle energy consumption using equations from the EPA for HEVs^38,39^, and for EVs, we use a multiplicative factor of 0.7, as suggested^40^. We refer the reader to the SI (Sects. 2, 3) for more details and a table with all key assumptions.

(iv) *Driving data* We use the United States Department of Agriculture Rural Urban Codes to classify our counties. Metropolitan counties were assumed to follow urban drive cycles (UDDS), rural counties followed highway drive cycles (HWFET), and the rest of the counties were assumed to follow a combined drive cycle (in the SI Sect. 2.2 for details on counties assignments).
(v) *Vehicle and battery manufacturing emissions* assumptions are summarized in the SI. We use an attributional framework for these estimates and assume that vehicle manufacturing emissions are constant across vehicle classes and fuel types. We consider NMC111 (LiNi_1/3_Mn_1/3_Co_1/3_O_2_) battery production in three locations (US, China, and Europe). We also consider LFP (Lithium Iron Phosphate, LiFePO_4_) produced in the US.
(vi) *Use-phase emissions* For gasoline, we include emissions from gasoline combustion from literature and include the upstream emissions of fuel production (extraction, processing, and transportation of gasoline)^24,41–43^. Our base case electricity emissions assumption for the vehicle charging assumes marginal emission factors for electricity generation (from Ref.^44^) and convenience charging. We also consider average emissions factors in our sensitivity analyses (from e-GRID) (SI Sect. 4)^45^) and a scenario where vehicles are charged during the lowest emitting hours (SI Fig. S9).

Following that, we estimate each county and vehicle’s critical emission factor that needs to be achieved so that BEVs reach lifecycle emissions parity with gasoline hybrid vehicles. When comparing CEFs to power sector generation emissions, we compare consequential (marginal)^44^ and attributional (average) emissions factors for each NERC region^46^.

We use upstream emissions associated with fuel extraction, refining, and transport for electricity from Tong and Azevedo^24^. This approach is suitable given that the upstream emissions from electricity account generally for less than 10% of the carbon intensity of electricity production (SI, Table S3). For gasoline/diesel, we use the upstream emissions associated with the extraction, refining, and fuel transportation from GREET^41^.

## Results and discussion

We use the traditional LCA assessment for different vehicles under simple scenarios and provide sensitivity analysis for some key assumptions. Then, we develop a new metric, the Critical Emissions Factor (CEF), which we define as the carbon intensity of the regional grid that would be needed for the lifecycle emissions from an electric vehicle to be at parity with the emissions of some of the most efficient gasoline vehicles in the market. We compare the critical emissions factors needed amongst conventional-electric vehicle pairs. We do so while accounting for regional and operational aspects that lead to different emissions levels in different locations, such as climate (ambient temperature) and driving conditions. We also compare CEFs to current grid emissions intensity in different U.S. regions to ascertain which regions have the highest GHG-reduction potential for the near-term widespread roll-out of electric vehicles. Inputs and results reflect 2018 conditions and are presented per unit distance traveled (1 km).

### Lifecycle CO_2_ emissions for different representative vehicles

**Figure 2:**
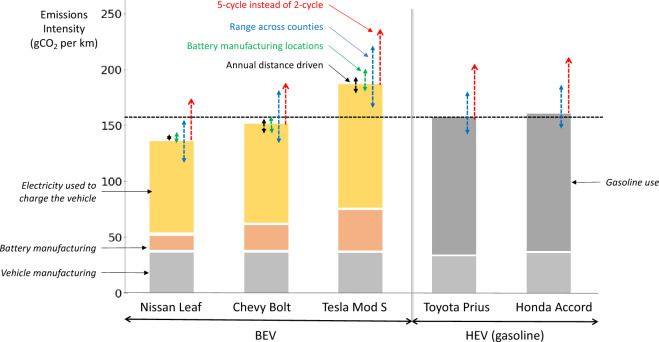
Life-cycle comparison between representative vehicles under different scenarios. The stacked represents a base-case scenario where: (i) electrified vehicles use NMC batteries manufactured in the U.S.; (ii) all vehicles drive 120 k miles; (iii) vehicles’ energy consumption accounts for ambient county level hourly temperature and county level drive cycles; (iv) vehicles are charged using convenience charging with an assumed emission factor of 597 gCO_2_/kWh; (v) values are weighted by total vehicle ownership in county. We highlight the contribution of different LCA phase emissions to the overall lifecycle emissions by displaying the emissions from vehicle manufacturing, battery manufacturing, and the emissions associated with charging/fueling the vehicles (including the upstream emissions from refining and transporting fuels). Red arrows show the effect of considering estimated 5-cycle energy use instead of 2-cycle; blue arrows show the range of emissions across counties; green arrows show the range of LCA emissions for each vehicle depending on battery manufacturing location; black arrows show the effect of different annual distance driven (ranging from 100 k miles to 150 k miles).

In Fig. 2, we show our estimates of life-cycle CO_2_ emissions. The stacked bars correspond to a base-case scenario where we assume the electrified vehicles use NMC batteries manufactured in the U.S. We also assume vehicles are driven 193,121 km (or 120 k miles^30^). The vehicles’ energy consumption accounts for ambient county-level hourly temperature and county-level drive cycles. We assume that the vehicles are charged using convenience charging. We also assume the emission factor for electricity when vehicles are charged is 597 gCO_2_/kWh, the average marginal emissions factor of electricity generated in 2018 in the United States for the hours the electric vehicles are charging^44^. Results are weighted by total vehicle ownership in the county from census data^47^ to account for differences across counties. Under these assumptions, we find that the Nissan Leaf and Chevrolet Bolt lead to the lowest life-cycle emissions, and the Tesla Model S has the largest emissions. Tesla Model S is a luxury sedan with lower vehicle efficiency (SI Table S1, S2) and a larger battery when compared to the other BEVs considered in this analysis. Other research, such as Kawamoto et al.^43^, find instead that EVs are lower-emitting than gasoline vehicles if vehicles are driven more than 60,779 km and assume higher emissions of battery manufacturing and chemistry than our study. Elgowainy et al.^48^ also finds that EVs with smaller range have the lowest carbon emissions, followed by HEV and that EVs with longer range have higher emissions.

This initial result shown in Fig. 2 does not fully capture regional differences and uncertainties. We thus add several scenarios, depicted as the vertical arrows in Fig. 2. Changing the assumed drive cycle from 2 to 5 (red arrows) increases emissions for all vehicles considered, but this change doesn’t impact the overall ordering of life-cycle emissions across our vehicles. The blue arrows show the range in LCA emissions for each vehicle across counties, stressing the importance of detailed geographical analysis. The green arrows highlight the implications of using batteries manufactured in different locations (NMC batteries produced in the U.S., China, and Europe^49^) and chemistries (NMC vs LFP produced in the US^41^). The differences reflect emissions associated with manufacturing the cathode, aluminum, battery management system, cell assembly, and other parts. We find that BEVs produced in Europe have the lowest LCA emissions. Chevrolet Bolt batteries manufactured in China could lead to life-cycle emissions similar to those of the gasoline HEVs considered. We also test the effect of different assumptions on vehicle miles driven, ranging from 100,000 miles (or 160,934 km) to 150,000 miles (241,401 km), and find that those do not significantly impact our normalized results.

**Figure 3:**
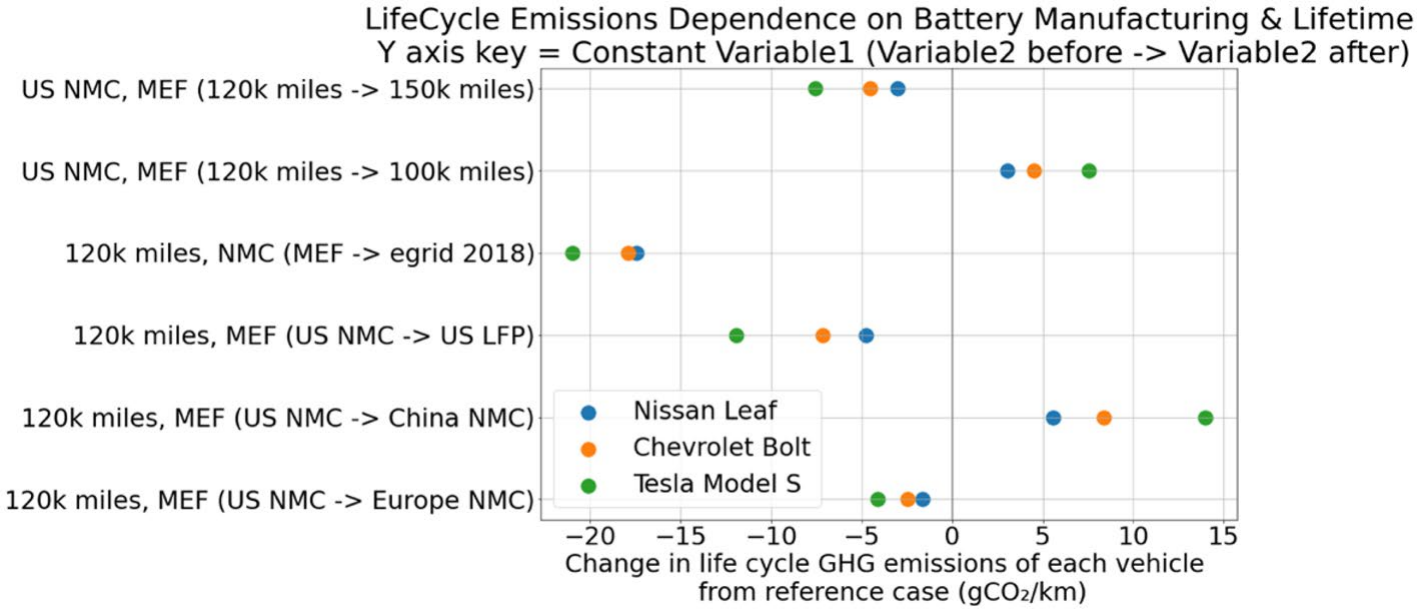
Sensitivity analysis for our representative vehicles’ life-cycle emissions change with changing input variables. Reference case input variables for each vehicle are lifetime = 120 k miles, battery manufacturing = US, battery chemistry = NMC111, and emission factors = marginal emissions factors.

Figure 3 provides a sensitivity analysis for life-cycle emissions of the BEVs as we change the assumptions regarding distance driven over the lifetime of the vehicle, location of battery production and its chemistry, and grid emissions factors used. The dots illustrate the effect of changing the assumptions inside the brackets while keeping other assumptions outside the bracket constant. For example, the topmost scenario (US NMC, MEF (120 k miles −  > 150 k miles) assumes the battery is US-produced NMC using marginal emissions factors for charging but testing for increasing total lifetime miles from 120 to 150 k miles. Doing so would decrease the life-cycle emissions of the Tesla Model S by 7 g CO_2_/km, Chevrolet Bolt by 5 g CO_2_/km, and Nissan Leaf by 4 g CO_2_/km. Increasing total lifetime, moving to cleaner locations of battery manufacturing, using domestically produced LFP batteries, and using average emissions factors reduces the life-cycle emissions intensity, and this effect is more pronounced for long-range (high battery capacity) electric vehicles than short-range (lower battery capacity).

### The lowest emitting vehicle differs by region

**Figure 4:**
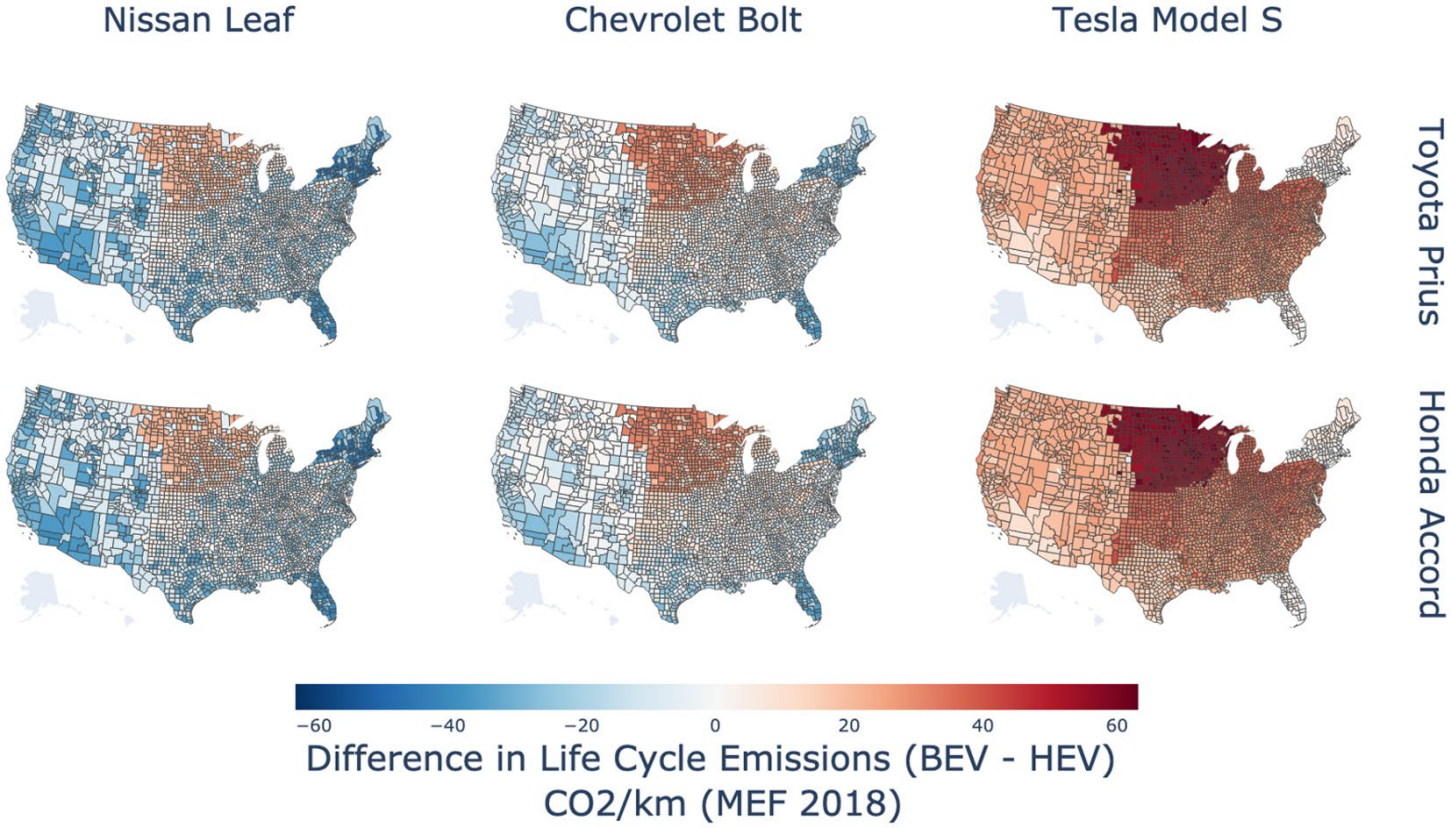
Difference between life-cycle CO_2_ emissions per km for battery electric vehicles and gasoline hybrid vehicles using hourly Marginal Emissions Factors for NERC regions in 2018 (Azevedo et al.^44^). Negative values (in blue) denote instances where battery electric vehicles are lower-emitting than gasoline hybrid vehicles. Positive numbers (in red) refer to values battery electric vehicles are higher emitting than gasoline hybrids. Vehicles are assumed to be driven for 120,000 miles over their lifetime and use convenience charging. The map was created by authors using Plotly for Python v5.9.0 https://plotly.com/python/.

Figure 4 expands on Fig. 2 by comparing the life-cycle emissions of BEVs to HEVs for each county under the same base-case assumptions detailed in Fig. 2. We find that Bolt and Leaf electric vehicles have lower emissions than the Prius and Accord hybrids in almost all counties of the West, Texas, Florida, and New England. In comparison, they have higher emissions in rural counties of the Midwest and the South. In contrast, the Tesla Model S has higher emissions than the Prius and Accord in all counties. We also ran the analysis considering average emissions factors (e-GRID 2018) (SI, Fig. S6), estimated 5-cycle fuel economy values, and charging during lowerst emitting hours and found that these produce estimates that are generally more favorable for BEVs (SI, Figs. S7, S8,S9). Compared to Yuksel et al.^14^, our findings are geographically consistent with higher BEV emissions in the Midwest and Southern U.S. but with lower emissions in the Western U.S. due to increased renewables.

### Achieving emissions reductions with electric vehicles beyond gasoline hybrids will require further decarbonization of the power grid in some parts of the United States

**Figure 5:**
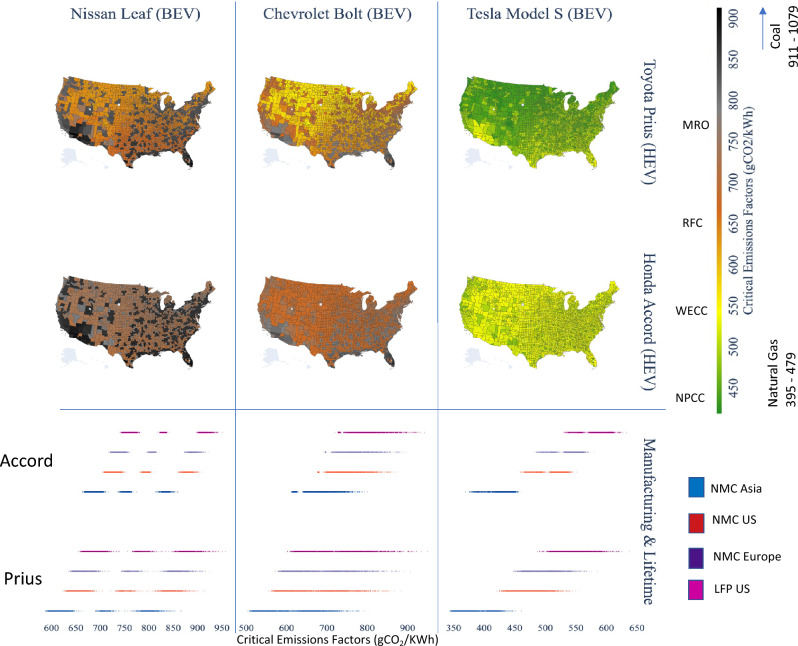
Critical emissions factors that the electric grid needs to achieve so that BEVs (Nissan Leaf, Chevy Bolt, or Tesla Model S) reach lifecycle GHG emissions parity with gasoline hybrids (Toyota Prius or Honda Accord Hybrid) assuming 120 k miles and NMC batteries manufactured in the US. Numbers adjacent to the color bar show the typical emissions intensity of different electricity generation sources (natural gas and coal power plants) and marginal emissions intensity for regional electricity grids for various NERC regions. The plots in the lower section illustrate the range of values for each county under different battery manufacturing locations and assumed battery chemistry. The map was created by authors using Plotly for Python v5.9.0 https://plotly.com/python/.

Figure 5 shows the critical emission factors needed for each BEV to reach greenhouse gas emissions parity with each gasoline hybrid. To achieve emissions parity with efficient gasoline hybrids, battery electric vehicles (US-produced NMC batteries) require power grid emissions lower than 421 to 915 gCO_2_/kWh, depending on the vehicle and location. Warmer areas in California and Arizona, areas bordering the Gulf of Mexico, as well as the southern states, and more urbanized areas on the West Coast, Appalachia, and East Coast have higher critical emissions factors, which indicates that it will make sense to move towards vehicle electrification for emissions reductions purposes even if the grid has a somewhat higher emission intensity. Colder and less urbanized parts of the country, such as the Great Planes, most of the Midwest, rural New England, and states tracing the Rocky Mountains, with a higher temperature dependence on vehicle dynamics, show lower CEF across comparative vehicles.

The color bar in Fig. 5 shows illustrative ranges of natural gas power and coal plant emissions (395–479 gCO_2_/kWh for natural gas^50^ and 911–1076 gCO_2_/kWh for coal) as well as regional marginal emissions factors of different NERC regions (NPCC/Northeast 450 gCO_2_ emissions per kWh or MRO/Midwest 773 gCO_2_ emissions per kWh^45^). Using the Tesla Model S as an emission reduction alternative will require low marginal emissions factors from the grid (with CEF values varying between 456 and 551 gCO_2_/kWh for the Honda Accord and between 421 and 556 gCO_2_/kWh for the Toyota Prius). These values are higher than the current average emissions intensity of United States’ electricity generation by EIA^51^ aggregated data as of 2021 (386 gCO_2_ emissions per kWh) and comparable to the current average combustion emissions intensity of natural gas power plants in the United States (395–479 gCO2/kWh), which means that natural gas-powered electricity would lead to Tesla Model S having lower emissions than the hybrid vehicles considered in this analysis.

Including variations for the location of battery manufacturing and chemistry, vehicle lifetime, and using derived 5-cycle fuel economy demonstrates that our results are largely in similar ranges but show variations based on assumptions. For instance, the leftmost plot under Leaf shows eight strip plots, four each for comparison with the Honda Accord Hybrid and Toyota Prius. Blue, purple, and red strips show the range of CEF with NMC batteries produced in Asia, Europe, and the US, while pink shows CEF with US-produced LFP, respectively. The sensitivity of CEF with battery location and chemistry depends on the battery’s size, with higher variations for Tesla–hybrid combinations and lower variations for Leaf and Bolt–hybrid combinations, but the overall ranges are similar. Additional sensitivity to derived 5-cycle fuel economy and lifetime miles is given in SI Fig. S11.

### Where do the marginal emissions from the grid need to be further reduced?

**Figure 6:**
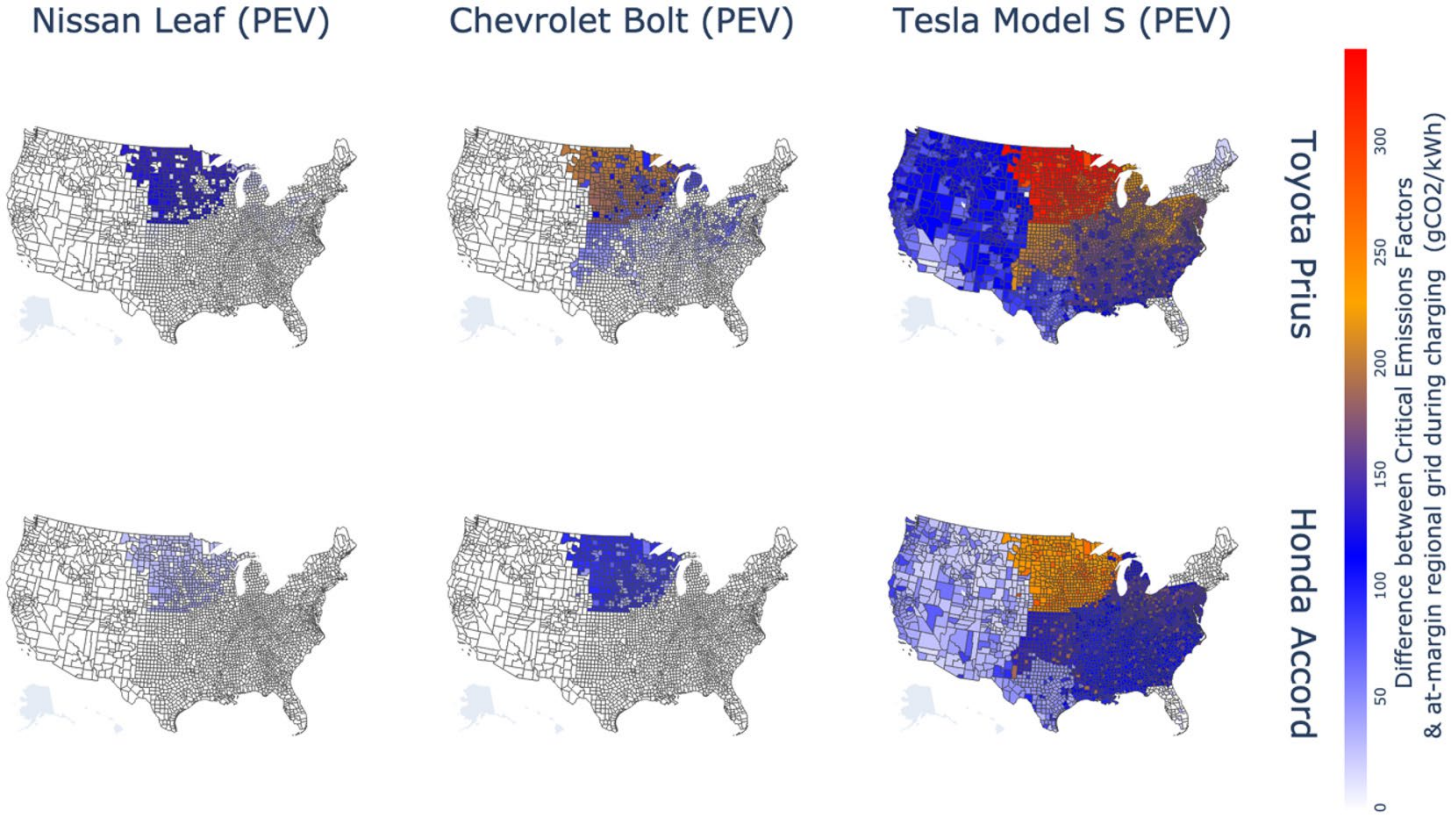
Reductions needed in current marginal grid intensity so that electric vehicles have less or the same consequential lifecycle emissions as gasoline hybrid vehicles. The figure assumes that NMC batteries are manufactured in the U.S. and that vehicles are driven for 120,000 miles over their lifetime. Areas shown in white identify places where the emissions from the grid are already equal to or lower than the critical emissions factor. The map was created by authors using Plotly for Python v5.9.0 https://plotly.com/python/.

In Fig. 6, we compare CEFs to current regional marginal emissions to identify which regions already have reached the required levels of local grid intensity to achieve carbon parity between battery electric and gasoline vehicles and which have yet to. We show the difference between CEFs and current marginal emissions factors for NERC regions weighted by the convenience charging profile (to reflect the charging time). In almost all parts of the United States, current marginal emission factors are already below CEF levels for the BEV Leaf and Bolt to have lower emissions than the gasoline hybrid Toyota Prius and Honda Accord. The marginal emissions factor must be reduced by 207 gCO_2_/kWh in the Midwest and parts of Appalachia for Leaf or Bolt to reach parity with gasoline hybrids. All regions of the US, except parts of the Northeast and Florida, would need to reduce marginal emission factors up to 342 gCO_2_/kWh for the Tesla Model S to have lower lifecycle emissions than these gasoline hybrids.

## Conclusions and policy implications

In this work, we present quantitative estimates of the reductions in power plant emissions needed in different regions across the U.S. while accounting for the effect of ambient temperature, drive cycle, battery manufacturing, battery chemistry, and lifetime on vehicle energy use. We underscore the importance of a synergistic opportunity for decarbonizing transportation as well as enabling a cleaner electricity grid in a spatially explicit manner. Our CEF estimates are intended to be applicable for both attributional and consequential frameworks, as they only find the breakeven emissions intensity when comparing two vehicle classes and are independent of marginal factors such as charging time, incremental generator, or electric vehicle fleet size. CEFs are functions of production emissions. Estimates of attributional production emissions can, in principle, differ from consequential production emissions, but we assume any such differences are negligible. We apply these CEF estimates to identify which regions have emission factors that achieve the CEF for specific vehicle comparisons, and we focus on a consequential framing, using 2018 marginal emission factors, because the consequential framing answers questions relevant to understanding the impact of changing EV adoption or EV policy.

Key climate change mitigation policy implications of our results are as follows. First, in some regions, choosing different types of electric vehicles can have important implications for the needed emissions reductions from the grid to ensure these vehicles reduce GHG emissions. Second, if combined cycle natural gas power plants are operating at the margin (or other electricity-generating technologies that have lower emissions factors than natural gas), a move to any of the electric vehicles considered in our analysis would generally reduce lifecycle emissions. These two points highlight that if vehicle electrification is considered a priority for policymakers as part of a climate mitigation strategy, coordination with grid decarbonization is necessary. Although our study used data specific to the United States, the framework applies to other regions globally. BEVs have higher greenhouse gas emissions than conventional vehicles in most parts of India based on the country’s current electricity composition, which is heavily dependent on coal^52^. In contrast, BEVs have 60–70% lower emissions than gasoline vehicles in Brazil^53^. Vehicle electrification benefits will depend on many place-based factors, but most importantly, the carbon intensity of the grid.

Third, while battery emissions are usually a small portion of the overall life-cycle emissions of electric vehicles, battery chemistry, manufacturing locations, and lifetime assumptions can impact the level of decarbonization needed in the electricity grid as the supply chain of electric vehicles becomes more diversified. For instance, the Tesla Model S requires about 40% lower critical emissions factors if battery manufacturing changes from domestic (U.S.) to Asia. Thus, understanding the trade-offs between domestic and international manufacturing locations for emissions and costs may be essential for future regulation assessment.

The intensity of driving and the assumptions on convenience charging are also necessary: increasing total lifetime from 100 to 150 k miles can increase the needed Critical Emissions Factors by 20% for Tesla-Prius parity, as the emissions of the vehicle and battery manufacturing are amortized over a larger number. Long-range (higher battery capacity) vehicles are more sensitive to these assumptions than low and medium-range electric vehicles. Changing from convenience charging assumptions to other modes of charging would have important implications for the emissions of electric vehicles since the marginal emissions vary throughout the day. We explore this sensitivity in SI Sect. 4 and Fig. S9.

Our study has several limitations: we do not include recycling and second life while calculating normalized lifecycle emissions, and we compare BEVs to some of the most efficient gasoline vehicles available rather than attempting to model the vehicles that BEVs are likely to displace (though we have also modeled scenarios where BEVs are compared to conventional ICE vehicles, as shown in the SI, Fig. S10). Our charging regimes are derived from NHTS 2017 data and can be made more heterogeneous across the U.S. with new data on charging behaviors and profiles. Finally, we focus here on greenhouse gas emissions. Additional analysis considering traditional air pollutants would be needed to assess the overall externality implications of electrification^7,23,24,54–56^.

### Supplementary Information

Supplementary Information.

## Data Availability

Most of the data generated or analyzed during this study are included in this published article and its supplementary information files. Hourly temperature data and NHTS-derived driving and charging profiles are available from the corresponding author upon reasonable request.
