# Supplementary material for: Ensuring greenhouse gas reductions from electric vehicles compared to hybrid gasoline vehicles requires a cleaner U.S. electricity grid
Source: Sci Rep. 2024 Jan 18;14:1639. doi: 10.1038/s41598-024-51697-1 (PMC10796910; doi:10.1038/s41598-024-51697-1)
Supplement: Supplementary file 1 — Supplementary Information. [file 41598_2024_51697_MOESM1_ESM.docx]

**Supplementary Document for**

**Ensuring greenhouse gas reductions from electric vehicles compared to gasoline vehicles requires a cleaner U.S. electricity grid.**

# Madalsa Singh^1^, Tugce Yuksel^2^, Jeremy Michalek^3^, Inês M.L. Azevedo^1,4,5,*^

^1^Department of Energy Science and Engineering, Stanford University, Stanford, U.S.A; ^2^Faculty of Engineering and Natural Sciences, Sabanci University, Istanbul, Turkey; ^3^Department of Mechanical Engineering and Department of Engineering and Public Policy, Carnegie Mellon University, Pittsburgh, U.S.A; ^4^Senior Fellow, Precourt Institute for Energy, Stanford University; ^5^Senior Fellow, Woods Institute for the Environment, Stanford University. *e-mail: [iazevedo@stanford.edu](mailto:iazevedo@stanford.edu)

Pages: 11

Figures: 10

Tables: 3

# Geographical Details

The U.S. electric power grid is interconnected, but to account for regional variation in power plant characteristics and fuel use, we assume the eight North American Electric Reliability Corporation (NERC) regions : FRCC, MRO, NPCC, RFC, SERC, SPP, TRE, and WECC [1] (**Figure S1**). Marginal Emissions Factors are estimated at the NERC region level.


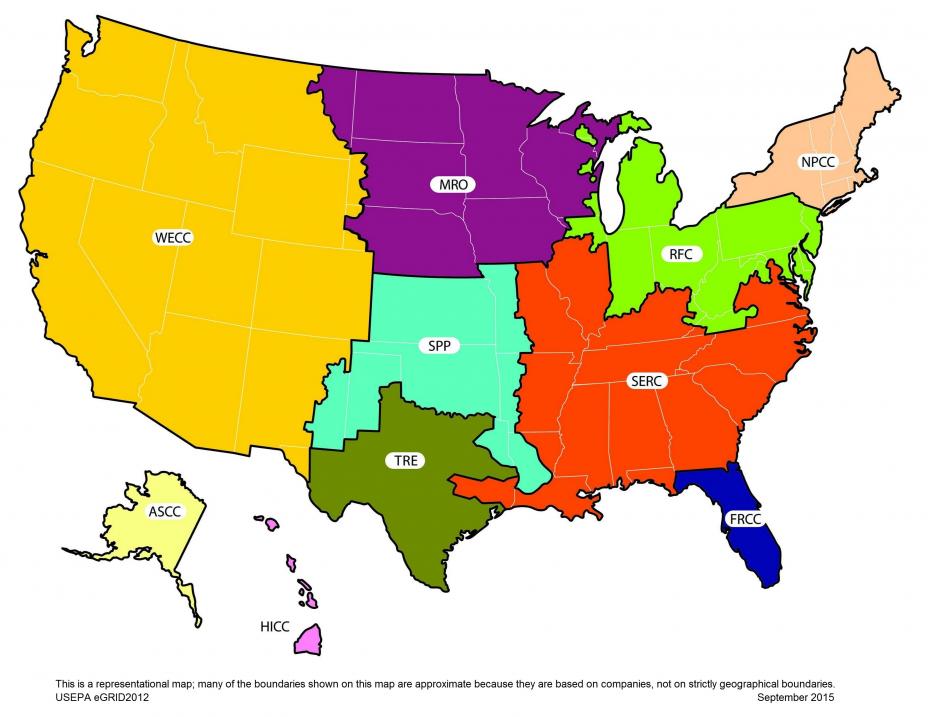


Figure S 1. Map of NERC regions in the U.S. The map was adapted by from EPA’s website: https://19january2017snapshot.epa.gov/energy/north-american-reliability-corporation-nerc-region-representational-map_.html

# Inputs

# 2.1 Regional Temperature

Annual average county-level temperature from NOAA’s Integrated Surface Database for 2018 [2].


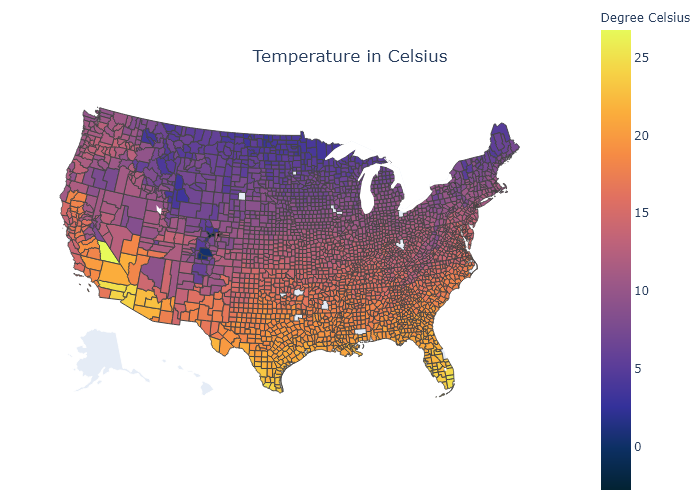


Figure S 2 County Level Average Temperature derived from NOAA Weather Stations in 2018. The map was created by authors using Plotly for Python v5.9.0 https://plotly.com/python/.

**2.2 Rural-Urban Continuum Codes**

The 2013 Rural-Urban Continuum Codes form a classification scheme that distinguishes metropolitan counties by the population size of their metro area, and nonmetropolitan counties by degree of urbanization and adjacency to a metro area[3].

Each county in the U.S. is assigned one of the nine codes listed below. Codes 6-9 are considered to be rural and assigned highway driving, while 1-3 are considered to be urban and assigned city driving, and 4-5 are assigned combined drive.

RUCC 1: Counties in metro areas of 1 million population or more

RUCC 2: Counties in metro areas of 250,000 to 1 million population

RUCC 3: Counties in metro areas of fewer than 250,000 population

RUCC 4: Population of 20,000 or more, adjacent to a metro area

RUCC 5: Population of 20,000 or more, not adjacent to a metro area

RUCC 6: Population of 2,500 to 19,999, adjacent to a metro area

RUCC 7: Population of 2,500 to 19,999, not adjacent to a metro area

RUCC 8: Less than 2,500 population, adjacent to a metro area

RUCC 9: Less than 2,500 urban population, not adjacent to a metro area


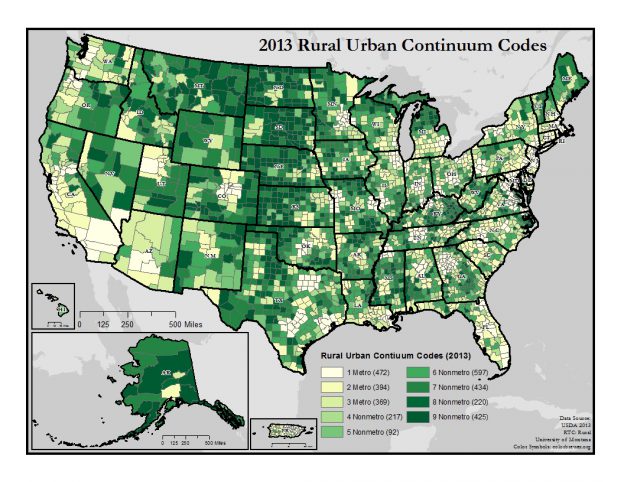


Figure S 3 RUCA Codes, USDA. The map adapted by the authors from the University of Montana’s Research and Training Center on Disability in Rural Communities. https://www.umt.edu/rural-disability-research/focus-areas/rural_disability/defining-rural.php

**2.3 Derived 5-cycle energy consumption**

To test for a more realistic performance of vehicles, approximations were used to calculate “real-world fuel economy” which is currently calculated through 5-cycle tests, which include drive cycles to reflect aggressive driving (US06) and driving with extreme temperatures (SC03) along with urban and highway driving to calculate the *actual* performance of vehicles on the road. Due to paucity of data for all test cycles at desired temperatures, we use regression equations with updated coefficients from 2017 for HEVs (gasoline hybrids) [4], [5] and a multiplicative factor of 0.7 for EVs [6] to find *derived* 5-cycle energy consumption data from 2-cycle dynamometer data for each temperature as given below. AAA provides both raw and corrected (with multiplicative factor) fuel consumption per mile values for EVs in the report. We also report 2-cycle fuel economy values from AAA and ANL dynamometer tests (Table S1) and derived temperature dependent 5-cycle fuel economy values in Table S2.

$$Five-cycle city fuel economy_{HEV}=\frac{1}{0.004091+\frac{1.1601}{UDDS fuel economy_{HEV}}}$$

$$Five-cycle highway fuel economy_{HEV}=\frac{1}{0.003191+\frac{1.2945}{UDDS fuel economy_{HEV}}}$$

$$Five-cycle city fuel economy_{HEV}=\frac{1}{\frac{0.43}{5-cycle city MPG_{HEV}}+\frac{0.57}{5-cycle highway MPG_{HEV}}}$$

$$5 cycle fuel econom{y \left( city or highway \right)}_{EV}= 0.7 X 2-cycle fuel economy_{EV}$$

| Model | Temperature | UDDS | HWFET | Combined |
| --- | --- | --- | --- | --- |
| 2018 Leaf (kWh/ mile) | -6 | 0.2632 | 0.2548 | 0.25942 |
|  | 24 | 0.1701 | 0.2086 | 0.187425 |
|  | 35 | 0.2023 | 0.224 | 0.212065 |
| 2017 Tesla Model S 75 D (kwh/mile) | -6 | 0.3815 | 0.2905 | 0.34055 |
|  | 24 | 0.2135 | 0.2121 | 0.21287 |
|  | 35 | 0.2674 | 0.2373 | 0.253855 |
| 2018 Chevy Bolt (kWh/mile) | -6 | 0.322 | 0.2695 | 0.298375 |
|  | 24 | 0.1554 | 0.196 | 0.17367 |
|  | 35 | 0.203 | 0.2156 | 0.20867 |
| 2015 Honda Accord Hybrid (gallon/mile) | -6 | 0.0239 | 0.0207 | 0.02246 |
|  | 24 | 0.016 | 0.0159 | 0.015955 |
|  | 35 | 0.0211 | 0.0173 | 0.01939 |
| 2010 Toyota Prius (gallon/mile) | -6 | 0.023 | 0.017 | 0.0203 |
|  | 24 | 0.015 | 0.0152 | 0.01509 |
|  | 35 | 0.0235 | 0.0199 | 0.02188 |

Table S 1: 2-cycle fuel economy with temperature from ANL and AAA dynamometer data

| Model | Temperature | UDDS HS1 | HWFET | Combined |
| --- | --- | --- | --- | --- |
| 2018 Leaf (kWh/mile) | -6 | 0.376 | 0.364 | 0.371 |
|  | 24 | 0.243 | 0.298 | 0.268 |
|  | 35 | 0.289 | 0.320 | 0.303 |
| 2017 Tesla Model S 75 D (kWh/mile) | -6 | 0.545 | 0.415 | 0.487 |
|  | 24 | 0.305 | 0.303 | 0.304 |
|  | 35 | 0.382 | 0.339 | 0.363 |
| 2018 Chevy Bolt (kWh/mile) | -6 | 0.460 | 0.385 | 0.426 |
|  | 24 | 0.222 | 0.280 | 0.248 |
|  | 35 | 0.290 | 0.308 | 0.298 |
| 2015 Honda Accord Hybrid (gallon/mile) | -6 | 0.032 | 0.030 | 0.031 |
|  | 24 | 0.023 | 0.024 | 0.023 |
|  | 35 | 0.029 | 0.026 | 0.027 |
| 2010 Toyota Prius (gallon/mile) | -6 | 0.031 | 0.025 | 0.028 |
|  | 24 | 0.021 | 0.023 | 0.022 |
|  | 35 | 0.031 | 0.029 | 0.030 |

Table S 2: Derived 5-cycle fuel economy from regression equations (HEV) and multiplicative factor (EVs

# Emissions Assumptions

- 1. **Emissions factors**

Vehicle and battery manufacturing emissions are taken from literature as noted in Table S3. These are attributional emissions associated with manufacturing a vehicle body and batteries in different locations using average emissions associated with processes and raw materials used in producing the output. Absent data on marginal consequential emissions, we use these estimates even though the scope of our LCA analysis is consequential. We have assumed that vehicle manufacturing emissions are constant across vehicle classes and fuel types, however other studies have concluded higher vehicle manufacturing emissions for EVs (7.3 – 9 tonnes) compared to internal combustion engine vehicles (6.9 -7.8 tonnes). Vehicle manufacturing emissions are one order of magnitude lower than the use-phase emissions and spread across lifetime wouldn’t impact the overall analysis [7].

Manufacturing emissions from producing the NMC111 ( LiNi_1/3_Mn_1/3_Co_1/3_O_2_) batteries in three different locations (US, China, and Europe) as well as changing battery chemistry to LFP (Lithium Iron Phosphate, LiFePO_4_) produced in US are used for analysis as noted in Table S3.

| **Emission Factors Input** | **Region** | **Emissions Intensity** | **Reference** |
| --- | --- | --- | --- |
| Gasoline Combustion | - | 8.89 kg CO_2_*/gallon* | EPA [42] |
| Gasoline Upstream | - | 1.75 kg CO_2_*/gallon* | GREET 2019 [43] |
| Vehicle Manufacturing | - | 37.5 gCO_2_*/km* | Hall and Lutsey [44] |
| Battery Manufacturing for NMC 111 | United States | 73 kg CO_2_*/kWh* | Kelly et al. [8] (Using GREET 2019) |
|  | Europe | 65 kg CO_2_*/kWh* |  |
|  | China | 100 kg CO_2_*/kWh* |  |
| Battery Manufacturing for LFP | United States | 50 kg CO_2_*/kWh* | GREET 2021 [9] |
| NERC level Average Marginal Emissions Factor, 2018 | FRCC | 484 CO_2_*/kWh* | Azevedo et al [10] |
|  | TRE | 576 CO_2_*/kWh* |  |
|  | WECC | 552 CO_2_*/kWh* |  |
|  | SPP | 666 CO_2_*/kWh* |  |
|  | MRO | 773 CO_2_*/kWh* |  |
|  | SERC | 625 CO_2_*/kWh* |  |
|  | RFC | 650 CO_2_*/kWh* |  |
|  | NPCC | 450 CO_2_*/kWh* |  |
| Upstream Electricity | FRCC | 48 CO_2_*/kWh* | Tong and Azevedo [25] and  Pehl et al [45] |
|  | MRO | 19 CO_2_*/kWh* |  |
|  | NPCC | 29 CO_2_*/kWh* |  |
|  | RFC | 24 CO_2_*/kWh* |  |
|  | SERC | 27. CO_2_*/kWh* |  |
|  | SPP | 31 CO_2_*/kWh* |  |
|  | TRE | 37 CO_2_*/kWh* |  |
|  | WECC | 27 CO_2_*/kWh* |  |
| e-GRID 2018 (NERC level Annual Average Emissions) | WECC | 350 CO_2_*/kWh* | [11] |
|  | FRCC | 422 CO_2_*/kWh* |  |
|  | MRO | 536 CO_2_*/kWh* |  |
|  | NPCC | 210 CO_2_*/kWh* |  |
|  | RFC | 472 CO_2_*/kWh* |  |
|  | SPP | 650 CO_2_*/kWh* |  |
|  | SERC | 451 CO_2_*/kWh* |  |
|  | TRE | 423 CO_2_*/kWh* |  |
| e-grid 2018 (US annual average emissions factor) | US | 476 CO_2_*/kWh* | [11] |
| e-GRID 2020 (US annual average emissions factor) | US | 406 CO_2_*/kWh* | [11] |
| US annual average MEF 2018 | US | 597 CO_2_*/kWh* | [10] |

Table S 3 : Emissions assumptions used in this study

- 1. **Comparison between Marginal and Average Emissions factors**

The distinction between marginal and average emissions factors is crucial when evaluating the emissions reduction potential of a potential action, such as adopting an EV or adopting EV policy. Over the last decade, average emissions factors, calculated as the total emissions produced in electricity sector over the total energy produced, have decreased by 30% in the United States as renewable penetration increases (black triangles line in figure S3). The highest decrease in average carbon intensity is seen in regional grid in Florida (FRCC) by 31%, while Midwest (MRO) has shown slower decrease of 6%. This is in stark contrast with regional and national Marginal Emissions Factors which have remained persistently high and increased in evening and night hours, which is when most people charge their vehicles (Figure S3). This increase in Marginal Emissions Factors is attributed to various reasons. Though renewables reduce average power sector emissions intensity, they rarely appear on the margin at today’s penetration levels (they are almost fully utilized regardless of small changes in electricity demand), and the presence of renewables shifts the dispatch curve, changing what mix of coal and natural gas appear on the margin at the times the electric vehicles charge. While coal use has been declining overall in the electricity consumption, coal units are becoming more responsive to marginal demand. Additionally, as these units increasingly operate at the margin, their utilization rates remain low and are typically inefficient in operations yielding higher marginal emissions [12].


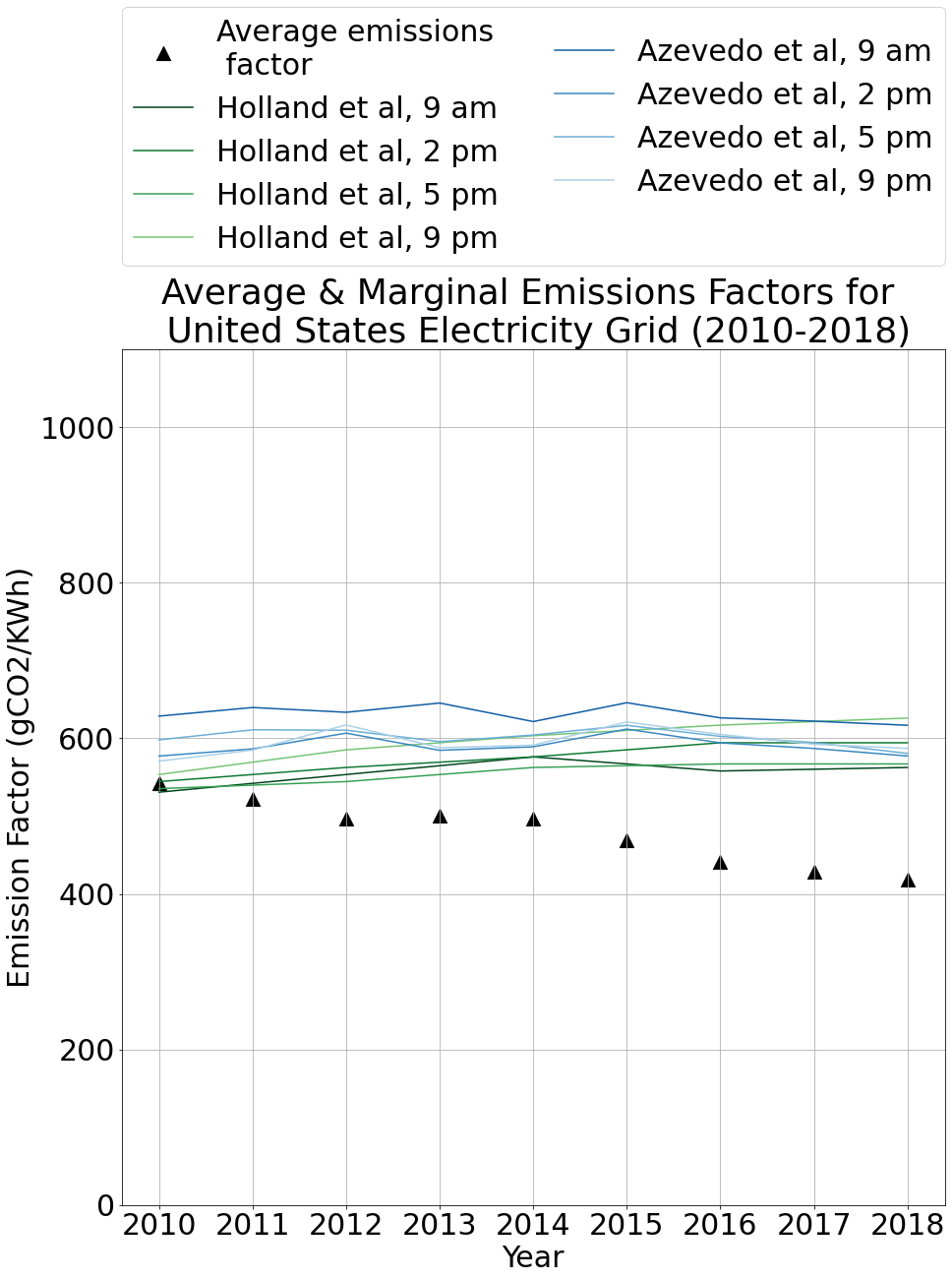


Figure S 4: US Average and Marginal Emissions Factors for specific hours in different years. From Azevedo et al [10] and Holland et al [12]

# Results

In this section, we present various intermediate results and sensitivities on inputs presented in the main text.


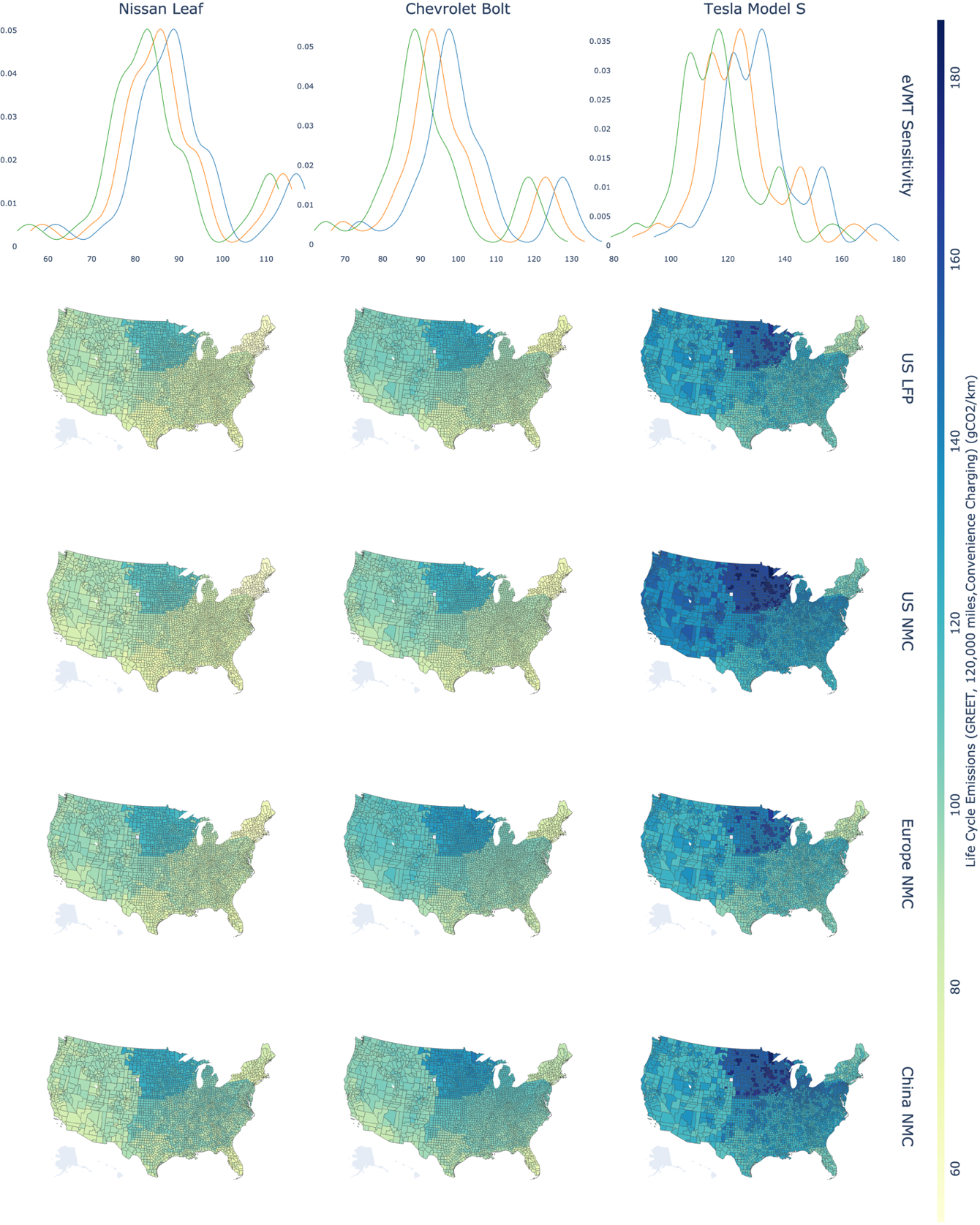


Figure S 5 : Life cycle emissions of Electric vehicles along with sensitivity of battery production location and lifetime. The map was created by authors using Plotly for Python v5.9.0 https://plotly.com/python/.

Figure S 6 : Difference between life-cycle CO_2_ emissions per km for battery electric vehicles and gasoline hybrid vehicles using Annual Average Emissions Factors for NERC regions in 2018 [11]. Negative values (in blue) denote instances where battery electric vehicles are lower emitting than gasoline hybrid vehicles. Positive numbers (in red) refer to values battery electric vehicles are higher emitting than gasoline hybrids. Vehicles are assumed to be driven for 120,000 miles over their lifetime and use convenience charging. Temperature dependent fuel efficiency values are 2-cycle. The map was created by authors using Plotly for Python v5.9.0 https://plotly.com/python/.


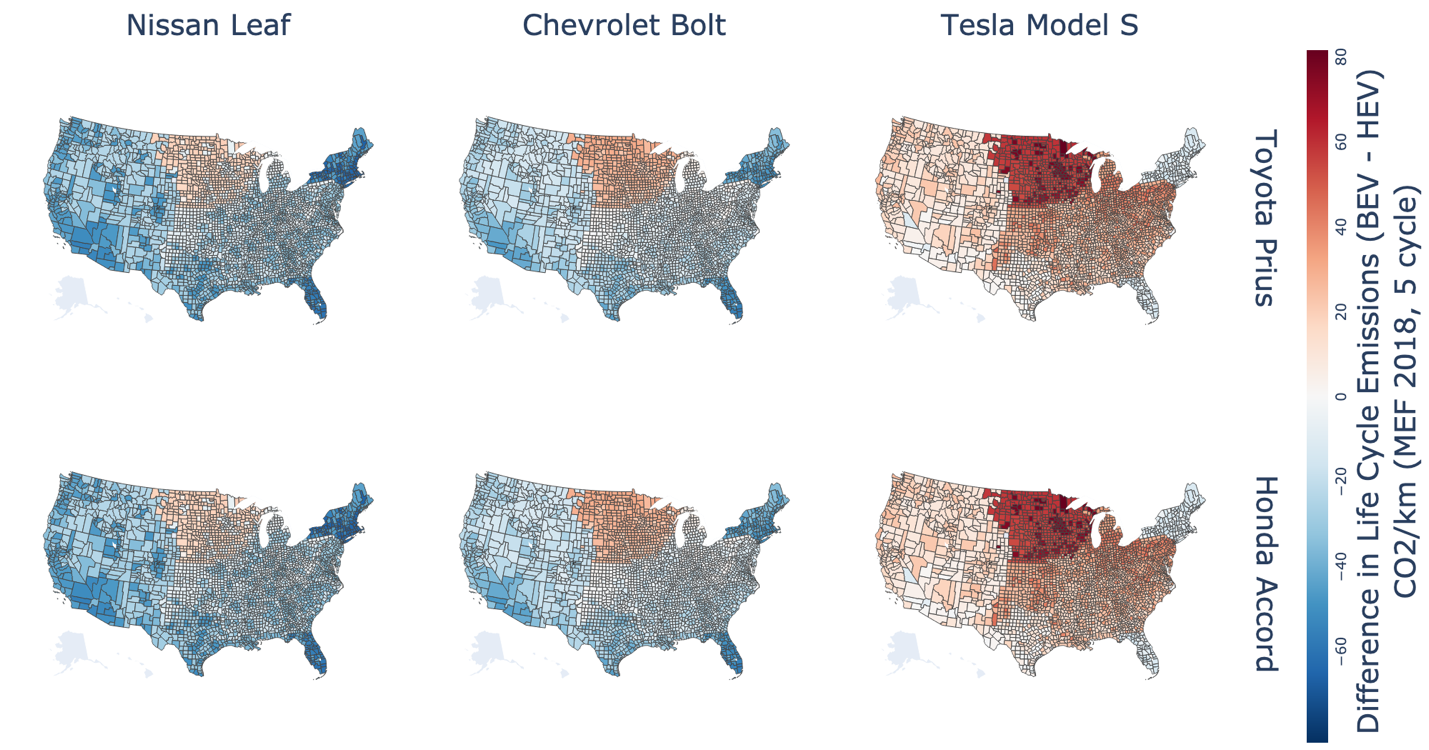


Figure S 7 : Difference between life-cycle CO_2_ emissions per km for battery electric vehicles and gasoline hybrid vehicles using Hourly Marginal Emissions Factors for NERC regions in 2018 [10]. Negative values (in blue) denote instances where battery electric vehicles are lower emitting than gasoline hybrid vehicles. Positive numbers (in red) refer to values battery electric vehicles are higher emitting than gasoline hybrids. Vehicles are assumed to be driven for 120,000 miles over their lifetime and use convenience charging. Temperature dependent fuel efficiency values are derived 5-cycle. The map was created by authors using Plotly for Python v5.9.0 https://plotly.com/python/.


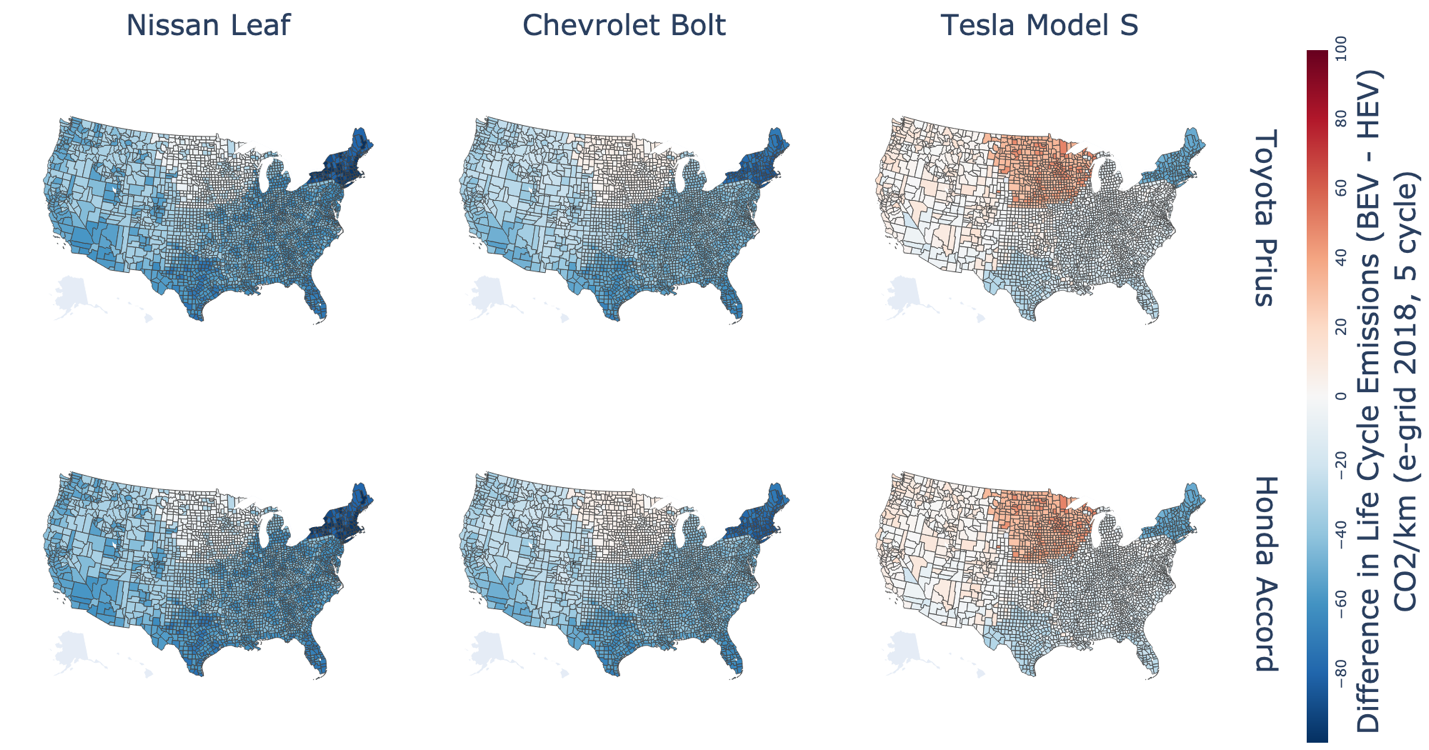


Figure S 8 Difference between life-cycle CO_2_ emissions per km for battery electric vehicles and gasoline hybrid vehicles using Annual Average Emissions Factors for NERC regions in 2018. Negative values (in blue) denote instances where battery electric vehicles are lower emitting than gasoline hybrid vehicles. Positive numbers (in red) refer to values battery electric vehicles are higher emitting than gasoline hybrids. Vehicles are assumed to be driven for 120,000 miles over their lifetime and use convenience charging. Temperature dependent fuel efficiency values are derived 5-cycle. The map was created by authors using Plotly for Python v5.9.0 https://plotly.com/python/.


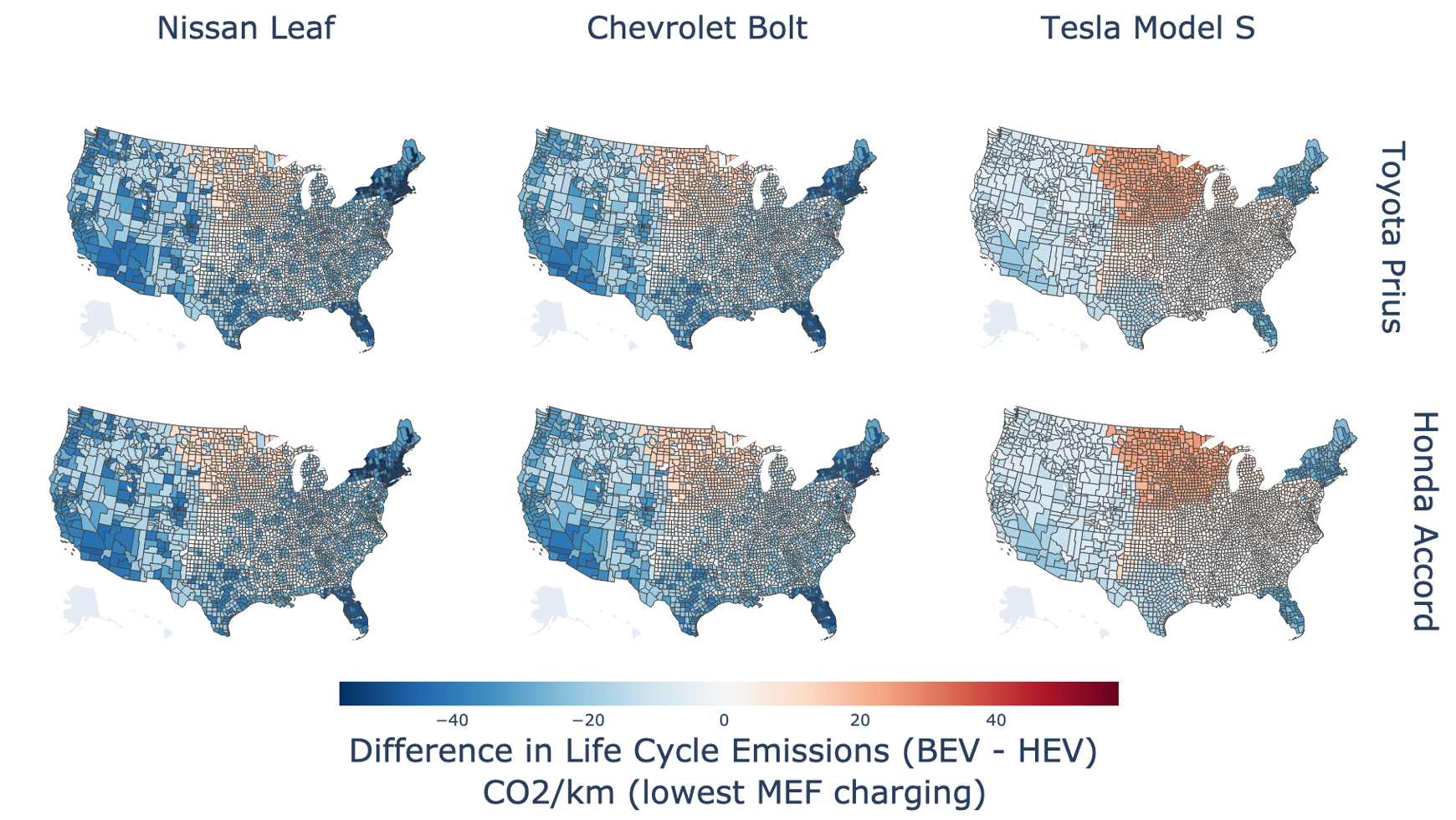


Figure S 9: Difference between life-cycle CO_2_ emissions per km for battery electric vehicles and gasoline hybrid vehicles with charging during hours with lowest Marginal Emissions Factors. Vehicles are assumed to be driven for 120,000 miles over their lifetime and Marginal Emissions Factors for NERC regions in 2018 (Azevedo et al [42]). The map was created by authors using Plotly for Python v5.9.0 https://plotly.com/python/.

In most regions, any types of electric vehicles will reduce emissions when compared to the Mazda CX 9 gasoline SUV. An exception is the Midwest, where there may not be emissions reductions. Tesla Model S is higher emitting than Mazda CX 9 by a maximum of 35 gCO_2_/km in parts of Midwest serviced by MRO.

Replacing a gasoline SUV with battery electric vehicle (third column) is desirable when regional at-the-margin grid intensity is between 597 and 1,552 gCO_2_/kWh depending on location. When comparing the critical emissions factors with the current marginal emissions factors we find that most regional grids, except in Midwest, have already reached a point where EV adoption leads to less emissions than the gasoline SUV. For long-range, heavier electric vehicle such as Tesla Model S, the grid emissions needs reductions by at most 164 gCO_2_/kWh to replace gasoline SUV in the Midwest.

In Figure S9, we assume that the vehicle is charged at the time where the marginal emissions factors are the lowest – thus providing lower bound emissions estimates for the charging of the vehicles. In this sensitivity analysis, we assume that the vehicle is charged at the time where the marginal emissions factors are the lowest – thus providing lower bound emissions estimates for the charging of the vehicles. This hour with the lowest emissions factors may or may not align with the middle of the day depending on the geographical location, but, regardless, it will provide a lower bound on emissions. Under such assumptions, we find that BEVs have lower emissions compared to HEVs in most parts of the country, except the Midwest for Bolt and Leaf, and the Midwest and South for Tesla. Optimizing charging for the lowest possible emissions factors the electricity grid can be a useful strategy to reduce emissions, but the spatial distributions of comparative emissions remain the same.


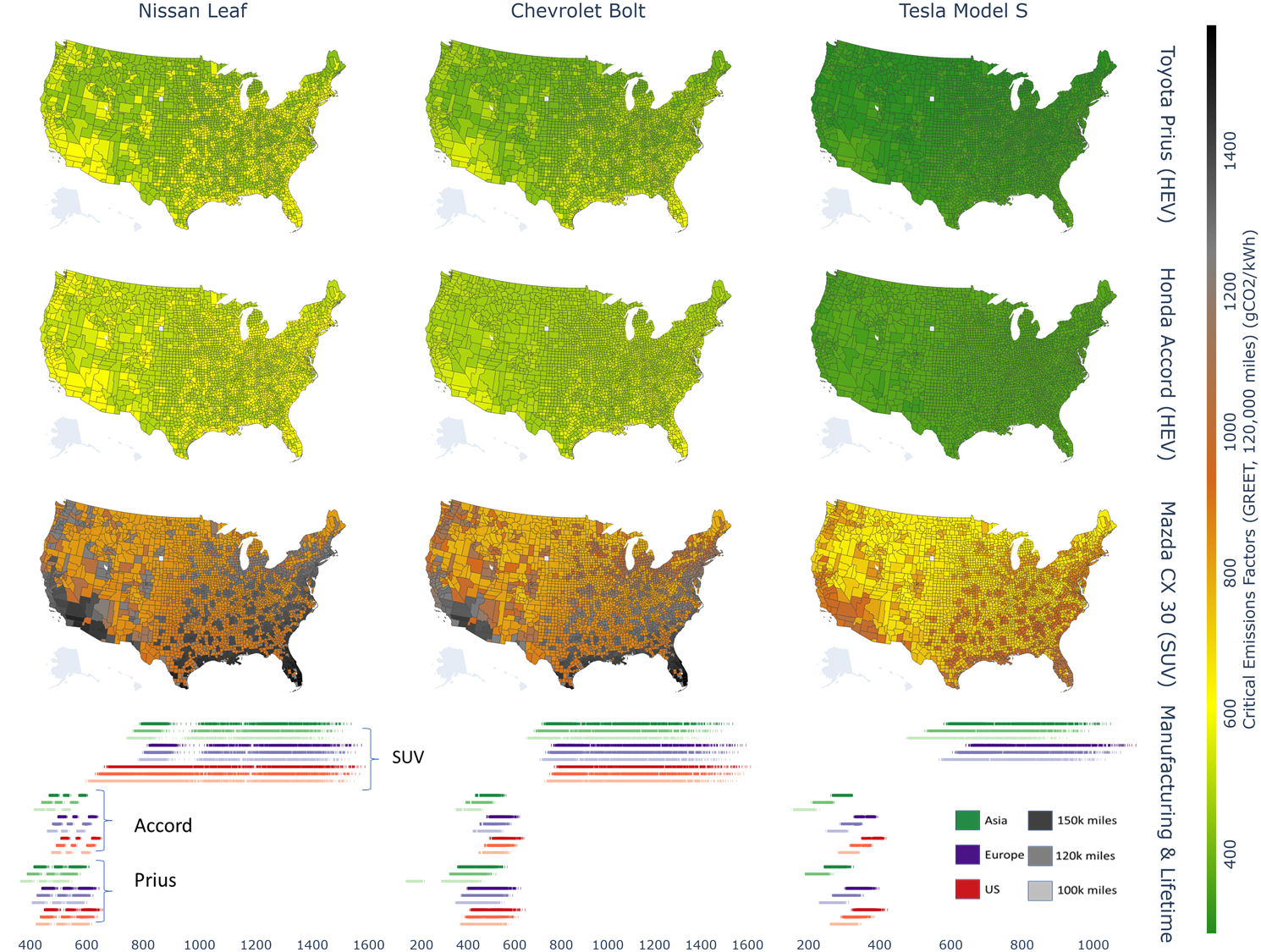


Figure S 10 Figure 6 Critical Emissions factors of EVs compared to hybrid gasoline vehicles and gasoline SUV (Mazda CX 30). The map was created by authors using Plotly for Python v5.9.0 https://plotly.com/python/.

Figure S 11 Critical Emissions Factors along with sensitivity on 5-cycle fuel economy and lifetime. The map was created by authors using Plotly for Python v5.9.0 https://plotly.com/python/.

[1] O. US EPA, “North American Reliability Corporation (NERC) region representational map.” https://19january2017snapshot.epa.gov/energy/north-american-reliability-corporation-nerc-region-representational-map (accessed Nov. 10, 2022).

[2] N. C. for E. Information (NCEI), “Integrated Surface Dataset (Global).” https://www.ncei.noaa.gov/access/metadata/landing-page/bin/iso?id=gov.noaa.ncdc:C00532 (accessed Oct. 13, 2022).

[3] “USDA ERS - Rural-Urban Commuting Area Codes.” https://www.ers.usda.gov/data-products/rural-urban-commuting-area-codes.aspx (accessed Jun. 15, 2022).

[4] “2017 and Later Model Year Light-Duty Vehicle Greenhouse Gas Emissions and Corporate Average Fuel Economy Standards,” *Federal Register*, Dec. 01, 2011. https://www.federalregister.gov/documents/2011/12/01/2011-30358/2017-and-later-model-year-light-duty-vehicle-greenhouse-gas-emissions-and-corporate-average-fuel (accessed Jul. 18, 2022).

[5] “40 CFR Part 600 -- Fuel Economy and Greenhouse Gas Exhaust Emissions of Motor Vehicles.” https://www.ecfr.gov/current/title-40/chapter-I/subchapter-Q/part-600 (accessed Jul. 18, 2022).

[6] “EPA test procedure for EVs-PHEVs, Nov 14, 2017.” [Online]. Available: https://www.fueleconomy.gov/feg/pdfs/EPA%20test%20procedure%20for%20EVs-PHEVs-11-14-2017.pdf

[7] A. Elgowainy *et al.*, “Current and Future United States Light-Duty Vehicle Pathways: Cradle-to-Grave Lifecycle Greenhouse Gas Emissions and Economic Assessment,” *Environ. Sci. Technol.*, vol. 52, no. 4, pp. 2392–2399, Feb. 2018, doi: 10.1021/acs.est.7b06006.

[8] J. C. Kelly, Q. Dai, and M. Wang, “Globally regional life cycle analysis of automotive lithium-ion nickel manganese cobalt batteries,” *Mitig Adapt Strateg Glob Change*, vol. 25, no. 3, pp. 371–396, Mar. 2020, doi: 10.1007/s11027-019-09869-2.

[9] “Argonne GREET Model.” https://greet.es.anl.gov/ (accessed Jan. 24, 2022).

[10] “Electricity Marginal Factors Estimates.” https://cedm.shinyapps.io/MarginalFactors/ (accessed Jun. 01, 2022).

[11] O. US EPA, “Emissions & Generation Resource Integrated Database (eGRID),” Jul. 27, 2020. https://www.epa.gov/egrid (accessed Jan. 24, 2022).

[12] S. P. Holland, M. J. Kotchen, A. J. Yates, and E. T. Mansur, “Why Are Marginal CO2 Emissions Increasing for U.S. Electricity? Estimates and Implications for Climate Policy,” p. 40.
